# A field guide for sampling bats (Chiroptera) for eco-epidemiological studies

**DOI:** 10.3389/fvets.2025.1605150

**Published:** 2025-09-09

**Authors:** Shariful Islam, Napoko Malika Kangoyé, Andrés Velasco-Villa, Abdoulaye Hama Diallo, Robab Katani, Luis E. Escobar

**Affiliations:** ^1^Department of Fish and Wildlife Conservation, Virginia Tech, Blacksburg, VA, United States; ^2^Global Change Center, Virginia Tech, Blacksburg, VA, United States; ^3^Center for Emerging Zoonotic and Arthropod-Borne Pathogens, Virginia Tech, Blacksburg, VA, United States; ^4^Department of Animals Biology and Physiology, Faculty of Earth and Life Sciences, University Joseph Ki-ZERBO, Ouagadougou, Burkina Faso; ^5^Independent Scholar, Suwanee, GA, United States; ^6^Department of Public Health, Faculty of Health Sciences, University Joseph Ki-ZERBO, Ouagadougou, Burkina Faso; ^7^Centre MURAZ Research Institute, MoH, Bobo-Dioulasso, Burkina Faso; ^8^The Huck Institute of the Life Sciences, Pennsylvania State University, University Park, PA, United States; ^9^Nelson Mandela African Institute of Science and Technology, Arusha, Tanzania

**Keywords:** bats, bat-borne, biosafety, biosecurity, Chiroptera, sampling, surveillance, welfare

## Abstract

Bats serve as reservoir hosts for numerous zoonotic pathogens of public health significance, including coronaviruses, lyssaviruses, and henipaviruses, while simultaneously playing critical roles in ecosystem function through pollination, seed dispersal, and pest control. The increasing frequency of bat-associated disease outbreaks has intensified research interest; yet standardized protocols for safe and effective bat sampling remain fragmented. We conducted a systematic review of bat sampling practices and synthesized comprehensive guidelines for capturing, handling, and sampling free-ranging bats for epidemiological surveillance and outbreak investigations. Our framework emphasizes three key elements, including (i) biosecurity measures to prevent pathogen spillover transmission from bats to humans, (ii) biosafety protocols to avoid spillback transmission, and (iii) welfare considerations to minimize the impact on bat populations. Through analysis of published literature and field protocols, we identified significant gaps between recommended and common practices in bat research. We present evidence-based recommendations for capture techniques, specimen collection, sample processing, and storage methods, with particular attention to maintaining sample quality while ensuring safety. Additionally, we provide detailed guidance for field laboratory setup, personnel training requirements, and emergency response procedures. The implementation of these standardized protocols will enhance the quality and compatibility of bat research data while protecting both human and bat health. This guide serves as a foundation for safe, ethical, and effective investigation of bat-borne pathogen epidemiology and ecology, particularly in resource-limited settings where disease emergence risks are often highest.

## 1 Introduction

Bat research is gaining interest due to the high abundance and diversity of bats globally, as well as their association with pathogens affecting human and domestic animal health (1, 2). Bats have unique physiological characteristics, such as high energy requirements and high body temperatures during flight (3–7). In comparison to other non-flying animals of similar size, bats have a longer lifespan than expected (8, 9). As flying mammals, bats possess unique features related to their physiological and ecological behavior, feeding and roosting habits, and long coevolutionary history with the viruses they carry (3, 9).

Bats have been identified as reservoir hosts for a growing number of emerging pathogens that cause diseases in humans, such as the Marburg virus, Nipah virus, Hendra virus, and rabies virus, just to name some of the most conspicuous viruses (2). The recent COVID-19 pandemic caused by the severe acute respiratory syndrome coronavirus 2 (SARS-CoV-2) is also considered to have an ancestral origin in bat coronaviruses (2, 10, 11). To date, thousands of bat-associated viruses have been discovered and classified under 28 viral families (2, 12). Nonetheless, researchers consider that knowledge about the bat virome is still limited and requires more comprehensive surveillance and monitoring of bat populations for pathogen discovery and characterization (2). Due to different ecological and anthropogenic changes, interactions between bats and humans are becoming more frequent and complex in nature, ultimately facilitating cross-species disease transmission (13). Aside from pathogen discovery and microbial eco-epidemiology studies in bats, research must focus on understanding their ecology and behavior, such as reproductive and foraging ecology, roosting patterns, and distribution (12, 14–16).

Conducting research on bats and bat-borne pathogens has an inherent risk of exposure to infectious agents for both researchers and bats (17). Personnel involved in research with bats and bat-borne diseases come from diverse disciplines, from the social to the life to the analytical sciences, which helps advance our understanding of the risk of bat-borne diseases. Nevertheless, a limited understanding of the biological risks when working with bats in the field may lead to the spread of pathogens between researchers and bats, posing a significant threat to public health and wildlife conservation (18). Improper handling and processing of bat-origin biological specimens may also generate inaccurate laboratory test results and misleading interpretations (19). Recent outbreaks of zoonotic diseases have also accelerated the development of biosafety guidelines and policies on bat research (17, 20).

Despite the intense interest in bat-borne infectious disease ecology and epidemiology research, field implementation of biosecurity and biosafety remains inherently challenging, posing significant hurdles to a comprehensive One Health approach (21). Logistical constraints are paramount for bat-borne virus discovery; accessing remote or difficult-to-reach bat habitats and deploying heavy equipment like harp traps in variable terrain can severely impede the efficiency and feasibility of large-scale and long-term studies. Furthermore, the coordination of adequately trained personnel is a challenge compounded by a lack of awareness or willingness among some researchers regarding best practices in challenging field conditions. Thus, logistical challenges can restrict the scale and frequency of sampling efforts. Overcoming these logistical and capacity barriers is crucial for understanding zoonotic pathogen dynamics and informing effective public health interventions (21).

**Figure 1:**
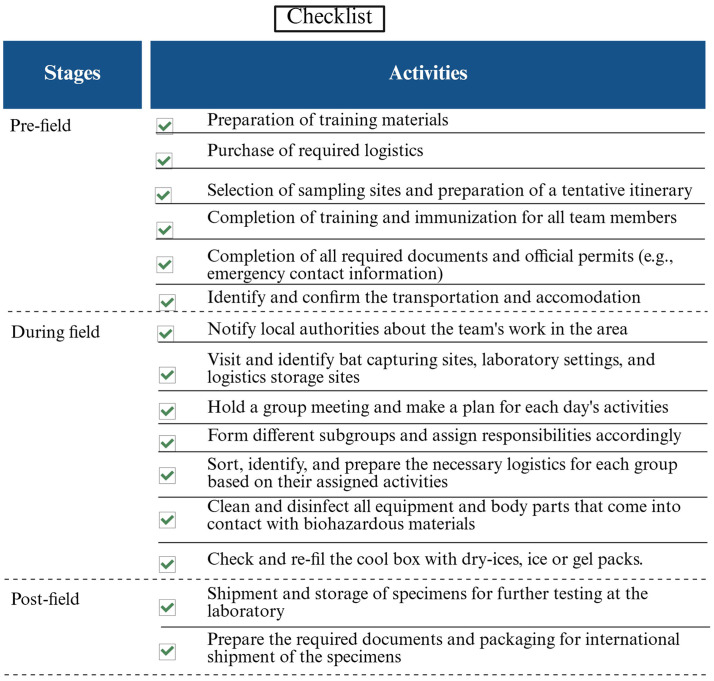
An operational flowchart of activities at different stages of bat research. Each activity is marked upon its completion to monitor and track research progress.

A key consideration in bat field research is the minimization of stress and enhancement of bat welfare. While essential for collecting valuable data, capture methods like mist-netting inherently carry the potential for stress, injury, or behavioral disturbance (22). For instance, prolonged entanglement in mist nets, improper handling of bats, or extended durations between capture and release after sampling can induce significant physiological stress in bats. Such stressors not only compromise animal welfare but can also impact the reliability of physiological data collected, highlighting the ethical imperative for continuous refinement of less invasive techniques and rigorous adherence to best-practice protocols (Figure 1) (22).

Research on bats should consider biosecurity and biosafety practices a priority from the conception of the research design to its implementation. Research projects should describe in full detail the study area, rationale for capturing, handling, and sampling techniques, and a description of field laboratories for sample processing. Data collection should be designed to identify and perform spatio-temporal follow-up of individual bats while guaranteeing their safe release into nature. There is, however, a lack of standard protocols and guidelines among field researchers to address biosecurity and biosafety practices when conducting research on different bat species across diverse habitats and interfaces globally. Without standardized guidelines for bat sampling, poor data quality and quantity could lead to inaccurate results or to research based on unethical approaches. This article provides guidelines for the capture, handling, and sampling of bats to reduce the risk of pathogen transmission between bats and humans and ensure the quality of samples (Figure 1). This review aims to effectively minimize the risk of pathogen transmission and maximize the scientific value of the samples and data collected while addressing basic ethical and conservation regulations. We provide examples from some well-studied bat species (Box 1) and offer an introductory tool for the personnel involved in future project design, grant proposals, project implementation, and laboratory sample processing in the research (Figure 1).

## 2 Pre-field preparation

### 2.1 General characteristics of bats

There are around 1,400 bat species grouped in 19 families in the Chiroptera order (23). Based on phylogenetic analysis, bats are currently grouped as Yinpterochiroptera and Yangochiroptera (24, 25). Bats differ from other mammalian species in their capacity to fly and use echolocation (22, 26). The places where bats live, mate, give birth to offspring, hibernate, rest, and protect themselves from adverse climatic conditions are called roosts (15). Bats can build roosts in sheltered areas (e.g., inside a cave or a building) and also externally (e.g., branches of a tree, tree cavities, or foliage) (9, 15). For instance, the common vampire bat (*Desmodus rotundus*) roosts in caves, house roofs, runways, tunnels, abandoned mines, wells, and hollow trees (27). In roosting sites, *D. rotundus* typically occupies the most elevated and darkest locations (28). Bats are known as nocturnal creatures; however, foraging times of bats vary among species. Some species leave the roost immediately or a bit late after sunset for foraging (e.g., *Pteropus medius*), whereas others leave the roost before sunset (e.g., *Nyctalus azoreum*) (29–32). Foraging time depends on weather, feeding behavior, and food preference (30, 31), and should inform sampling design. Bats have significantly different reproductive strategies than other mammalian species, which increases successful births and the fitness of both the mother and offspring in terms of lifespan, reproduction, and mortality rate (14, 16). The reproduction of bats can be characterized as multiple reproductive events with a delayed onset of sexual maturity and low litter size. With a few exceptions, bats usually produce only one offspring in each gestation (16).

### 2.2 Study design, sample size, and species consideration

Research requiring bat handling needs a meticulous sampling design that accounts for site selection, a statistically robust sample size, and sampling bias mitigation (33). Inadequate mitigation of biases in sampling design can lead to difficulties in data analysis, potentially misleading the performance of analytical models and interpretation (34). To mitigate sampling bias, study areas could be stratified into different sampling sites based on habitat type to account for the potential effect of environmental configuration as a confounding factor. Considering the distance among sampling points could help increase spatial independence among bat roosting sites when such information is needed (35). Studies should account for data requirements of the expected analytical method, logistics in the study area, study duration, species population dynamics, and budget (36).

Because bats are small, nocturnal, cryptic, and conspicuous when active, estimating the sampling size of bats is challenging (37). The objectives of the study, target species, capture approach, and data precision needed all play a role in establishing the sample size for bat surveys. Based on the study design, power analysis, and simulation studies aid in providing more precise sample size estimations (37–40). For some research questions, the adequate sample size and duration of the research project may not be viable. Thus, researchers should plan to generate alternative approaches, such as collecting a pool of environmental samples from the bat roosts, developing models or simulations, or exploring complementary data and sample repositories.

BOX 1 **Quadripartite of animal health research, including personnel safety (human health), animal health and welfare, environmental health, and sample quality.**

*Introduction:* We explored the general use of protective equipment to prevent pathogen spillover from and spillback to bats. We focused on *D. rotundus* a vampire bat species frequently handled for pathogen detection studies in Latin America.

*Methods:* We conducted a scoping review to know the different techniques used to capture *D. rotundus*, their explanation in the paper and what kinds of biosecurity and biosafety protective measures were used. We searched on Web of Science using the term “vampire bat” in all fields on 23 February 2023, and found 562 papers (Supplementary material S13). To determine eligibility for inclusion, we reviewed the abstract and materials and methods sections of each paper. Our selection of papers for this review was guided by specific criteria. Inclusion criteria mandated that papers focus exclusively on vampire bats, utilize methods of active capture and sampling for these bats, be published in English, and have their full text publicly available. Conversely, studies were excluded if they did not involve vampire bats, lacked active capture and sampling methodologies, were not written in English, or did not have their full text publicly available (Supplementary material S13). From the selected papers, we extracted information on bat-capturing techniques used, and implemented biosecurity and biosafety practices.

*Results:* Out of 449 articles found, 161 (35.86%) described the sampling collection methods (Figure IA). Researchers used 16 different combinations of bat-capturing techniques during their field sampling, where the highest (42.86%) number of the studies used solely mist net or in combination with other techniques (Figure IC). A percentage of 44.7% articles did not mention their sampling techniques (Figure IB). Only 36.02% of articles briefly explained their net or trap setting techniques and where they set the net or trap. Only 2.48% (n = 4) articles mentioned biosafety and biosecurity tools, especially the gloves and masks they have used during their bat sampling (Figure ID).

*Discussion:* In an open areas researchers tend to use mist nets (95, 96), whereas in caves or at opening of caves harp trap or combination of harp trap and hand nets are used (97–97). We found that researchers commonly mentioned that they captured *D. rotundus* but did not name the techniques they used (100–97). So, single or combination of more than one technique are commonly used for *D. rotundus* capturing.

Based on the results of the mini review, there is a limitation to not following the correct biosafety and biosecurity processes. Alternatively, researchers may be reluctant to share the specifics of the biosafety and biosecurity procedures they followed during their study process or found such information irrelevant for the manuscript. None of the papers revised explained the protective tools used. Articles generally used the term “biosafety” or the use of masks or gloves (103–97).

**Figure:**
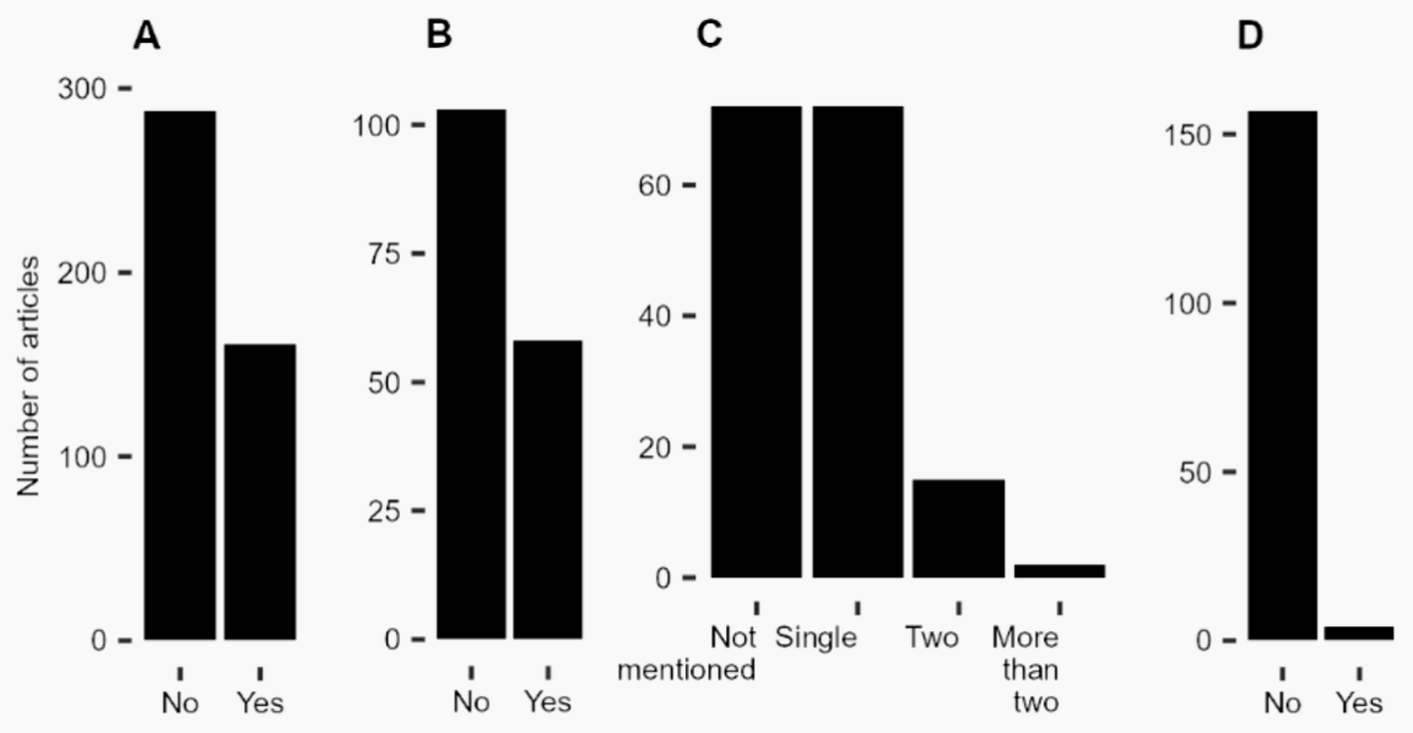
FIGURE I Findings from the scoping review on *Desmodus rotundus*. **(A)** Articles describing the methods used to collect data on *D. rotundus.* **(B)** Articles describing the methods used to capture *D. rotundus*. **(C)** Number of sampling techniques used to capture *D. rotundus*. **(D)** Articles with information related to biosecurity.

### 2.3 Training

Every team member (i.e., researcher, student, or technician) participating in bat sampling should complete training requirements to engage in the field activities related to bat research. Every research organization or academic institute should have its own policies and rules regarding research training. For example, training is generally available through the Institutional Animal Care and Use Committee (IACUC), occupational health, and environmental health and safety offices, among others. In the absence of institutional training opportunities, the team leads or principal investigators can organize training sessions to educate project members about biosecurity and biosafety practices at different stages of the research project. It is the responsibility of both team leaders and members to complete the minimum training required to implement biosecurity and biosafety measures to reduce harm to humans and bats. The team lead can follow an operational risk assessment matrix to evaluate the knowledge of team members and take appropriate action (Supplementary material S1).

### 2.4 Compilation of required documents

During the implementation stages of bat research, local wildlife, health, or environmental protection agencies may require certain documents to be completed before sampling can be initiated. The team should have copies of these documents in the field to access when there is limited internet access or even a lack of laptop devices. For example, local police or rangers may ask the team to show permit documents for bat sampling. Furthermore, it is suggested to carry the data collection form or survey questionnaire to be used to collect data from the field. In summary, the field team should bear copies of critical documents during fieldwork.

- Study protocols (short summary of the research project proposal in English and the local language).
- Approval of the study protocol by the relevant NHEC/Institutional Review Board (IRB) or any other regulatory authorities.
- National and international research permits to capture and sample bats from the study area.
- Data sheet or survey questionnaire for information compilation.
- Immunization records and respiratory fit test cards of team members.
- Travel documents (e.g., passport, visa, air tickets, and emergency contacts).

### 2.5 Fieldwork logistics

Protocols for data collection, biosecurity and biosafety plan, and sample storage are fundamental for successful field expeditions. Bat sampling sites are generally located in remote areas. For international fieldwork, it may be challenging to find the necessary supplies or equipment at study sites. Therefore, it is important to learn about the target bat species and study location to purchase supplies, materials, and equipment well in advance. Broadly, equipment, materials, and supplies necessary during the sampling can be classified into two groups: for capturing bats and for sampling bats. The logistics for capturing and sampling bats should cover Personal Protective Equipment (PPE), instruments, materials and consumables, processing station (i.e., portable tables and chairs), and storage (e.g., tanks and coolers). Tasks can be organized by teams; for example, the bat capturing team could focus on purchasing mist nets and traps. Additionally, a portable centrifuge machine with batteries and cables is necessary to separate serum from blood samples. For chemical restraint and euthanizing the bats, the team needs to procure all necessary chemicals. In some cases, locally made utensils for net setting have been found successful for the easy execution of bat capturing. For example, a modified form of mist net is made locally to capture *Pteropus* bats in Bangladesh, and researchers are utilizing that successfully for captures (41). As for pre-field logistics, team members should focus on making risk assessments to determine which vaccines or pre-exposure medications the team members may require (e.g., rabies, yellow fever, and malaria treatment), as well as other post-exposure or palliative drugs (first aid kit) to address any field incident.

### 2.6 Biosecurity and biosafety

To control and prevent the spread of zoonotic diseases, it is crucial to implement proper biosecurity and biosafety precautions during interactions with animals (42). During fieldwork, biosecurity aims to reduce pathogen transmission from wildlife to humans, while biosafety refers to efforts to reduce pathogen transmission from humans to wildlife. Biosecurity has been defined elsewhere as the measures to avoid the transmission of animal pathogens or parasites to the general public and the environment by using containment equipment (42). Biosafety has been defined as the set of measures taken when handling biological material to prevent the introduction of novel pathogens into new populations (43). Both biosecurity and biosafety require the use of PPE.

During fieldwork, team members need PPE, which includes respirators, gloves, face shields, coveralls, dedicated long-sleeve clothing, gowns, and goggles, as standard infection control precautions (20). PPE in bat-borne disease research creates barriers to protect personnel and bats from pathogens and hazardous materials through various entry points like skin, mouth, nose, and eyes (44). The necessary PPE equipment depends on the biological agents being handled and the research settings (Table 1) (Supplementary material S2). During the removal of PPE, researchers should take extra precautions as all the PPE is assumed to be contaminated and there is a chance of exposure to contaminants. Therefore, we suggest a step-by-step sequence to decontaminate and remove the PPE after completion of designated work (Supplementary material S3).

**Table 1:** Minimum personal protection equipment required to perform bat capturing and sampling at field level at different settings.

Purpose	Settings	Works involved	PPE required	References
Basic research (e.g., viral ecology)	Cave or abandoned building	Bat capturing and sample collection	Coverall with hood N95 or P100 respirator Protective eyeglasses Powder-free nitrile gloves	Kingston et al. (20)
	Bat roost or foraging sites	Environmental samples	Coverall with hood N95 or P100 respirator Protective eyeglasses Powder-free nitrile gloves	Kingston et al. (20)
	Bat roost or foraging sites	Individual bat capturing	Dedicated long sleeve clothes or coverall with hood. N95 or P100 respirator Protective eyeglasses Powder-free nitrile gloves	Kingston et al. (20)
Applied research (e.g., outbreak investigation)	Bat roost or abandoned building	Environmental sampling	Coverall with hood N95 or P100 respirator Protective eyeglasses Powder-free nitrile gloves	Epstein et al. (41)
	Bat roost or abandoned building or foraging sites	Individual bat capturing	Coverall with hood N95 or P100 respirator Protective eyeglasses Powder-free nitrile gloves	Epstein et al. (41)

To prevent snake bites and leg injuries during fieldwork, researchers should wear gumboots or waterproof shoes in addition to their personal protective equipment PPE mentioned below. Researchers should use shoe covers to prevent the transfer of dirt, debris, and contaminants from footwear into clean or sterile areas, such as laboratories, operating rooms, or food processing facilities. Researchers use head covers to prevent hair shedding and contamination. Researchers should use face shields over the mask to take extra protective measures against airborne droplets.

### 2.7 Risk management in the field

Beyond the use of PPE to reduce health risks, effective risk management should include diversity, equity, and inclusion (DEI) practices, which can help increase the well-being and safety of the team in field sites. DEI practices, however, require a basic understanding of the team and local culture and may involve previous training to take the necessary steps to prevent, prepare for, and respond to risks.

#### 2.7.1 Diversity, equity, and inclusion (DEI) in research related to bats

Conducting fieldwork may be a lifelong opportunity to gain exposure to multiple cultures and landscapes and to acquire research experience (45). For some team members, getting a chance to engage in active fieldwork research is a rare opportunity, especially for those from underrepresented groups and low-income backgrounds. Some research institutions are working to create a more diverse, fair, inclusive, and accessible environment (45). There are no definitions of DEI that fit across-the-board (46); a particular research organization, department, or laboratory should identify its own definitions and guiding principles that consider what is effective for them. Generally, diversity is the presence of differences in backgrounds among team members, resulting in multiple identities and perspectives such as nationality, gender, ethnicity, religion, race, and sexual orientation (46, 47). Equity is the provision of equal opportunities for all team members to grow, contribute, and develop in achieving goals irrespective of their identity (46). Inclusion is a continuous effort to engage in a team that ensures authentic involvement, empowered participation, and fosters a sense of belonging (46–48). Implementing DEI principles based on gender, geographic, and socioeconomic backgrounds to conduct research would provide opportunities for underrepresented members to gain insights into the bat sampling process in different settings (e.g., cultural rules, race, religion, or ethnicity). Underrepresented communities possess rich indigenous knowledge, but individuals from marginalized groups are still underrepresented in health research (49). To effectively practice and implement DEI principles during field expeditions, team leaders can recruit a diverse cohort of team members, provide training, recognize contributions, foster an inclusive environment where all voices are valued and heard, articulate common objectives, treat team members with respect, establish mentorship programs, assess anti-discrimination policies, ensure open communication, and promote collaborative research initiatives (50). Small efforts make the difference. For example, first aid kits must include menstrual hygiene products to recognize and validate the presence and needs of female researchers, making fieldwork a more inclusive environment for all (107). Implementing DEI principles would be an effective approach for more innovative and creative bat research and for promoting capacity building to prevent zoonotic diseases and foster bat conservation.

#### 2.7.2 Communication

##### 2.7.2.1 Local/national

The team leader should share fieldwork updates with the team, including where and when the team will be working, bats captured and sampled, and adaptive management or changes in the plans. Members should have information about emergency support regarding local hospital facilities and police, waste disposal, and alternative short-term storage of reagents and samples in case of emergencies. Community leaders can help researchers ensure access and smooth operations in the field. At the end of the study, team leaders should share executive summaries in plain language of the research conducted with local stakeholders.

##### 2.7.2.2 International

The team may need to share overall fieldwork updates with their home institution. This update may include a list of products and challenges that required modification to the plan, as well as summaries of discussions, conclusions, and recommendations reached. The institution should know the location of the team during the duration of the expedition.

### 2.8 Handling common incidents

In a remote area, an unwanted situation may arise. For example, team members may suffer unexpected injuries while in the field. For the fast and proper management of any incident, researchers should gather and organize the addresses and contact information of local police stations, sample collection permitting authorities, and acquire detailed knowledge of the fastest evacuation routes and secure transportation to reach the nearest hospital or healthcare centers rapidly by effectively engaging with local stakeholders and emergency support organizations. Based on incidents and the surrounding environment, we provided a protocol to follow in emergency situations (Supplementary materials S4, S5).

### 2.9 Transport

To reach field sites and carry the logistics, the team may need to travel to local, national, or international areas. Transportation arrangements are necessary before the expedition starts. We can broadly classify the transport arrangements into two groups: short-term long distance (e.g., another state or country) transportation and long-term short distance transportation (e.g., systematic surveillance of a colony requiring continuous storage of samples and supplies), so that the necessary vehicles should be secured before the expedition.

## 3 Field operations

### 3.1 Bat-capturing methods

Researchers use various techniques to capture bats, including mist nets, harp traps, hand nets, and direct hand capturing of different bat species (51, 52). These capture methods have both pros and cons. Researchers must decide which is most suitable and available for their research purposes. Sometimes, researchers may use a combination of different techniques based on the bat-capturing sites and bat species of interest. For instance, if a study requires a specific bat species from caves and foraging areas, researchers can use mist nets at foraging sites and cone traps at roosting sites. To capture small bats, a group of two people should be enough to set the net. Nevertheless, if the mist net is large (e.g., 20 ft), more than two people may be required in areas with high bat activity. Net-setting techniques and logistics may differ (height and length of the net, pocket size of the net, etc.) based on the study location and target species (Supplementary material S6). Here, we describe some capture techniques that have been used for different bat research purposes.

#### 3.1.1 Mist net

Mist nets are used globally to capture bats and are typically made of polyester and nylon with thicker threading (53). Every bat species differs in terms of body size and flyway height above the ground. The mesh size and height of the mist net setting are determined based on the target species of bats. For example, capturing bats of the Pteropodidae family requires a bigger mesh size mist net than capturing bats of the Megadermatidae family. Choosing an ideal mist net influences bat capturing success (54). Mist nets are selected based on the characteristics of the surrounding areas of the roost (55). Researchers should have a general idea of the bats’ flight paths before selecting netting sites. To explore bat flyways, researchers can visit the net setting sites at least 30–40 min before the species generally becomes active and identify sites with likely success based on early captures (56). Nets can be suspended between bamboo or long, straight trees, or commercially available aluminum poles or polyvinyl chloride (PVC) pipes using ropes. It may be necessary to be cautious when using the aluminum pipes to hang the mist net, because ropes may slip and reduce the shelf size. Mist nets allow researchers to capture bats at their roosting or foraging sites (51). In adverse weather conditions (e.g., windy weather, heavy rain), mist nets need to be kept closed; otherwise, nets may become messed up, which is sometimes difficult to fix.

The exit of bats from their roosting sites varies based on the species and is associated with the time of sunset. Other factors, such as weather and the presence of insects, might or might not influence bat foraging time, but there is no convincing evidence available (55). In the case of *Tadarida brasiliensis mexicana*, a common species in the Americas, the time to exit from the roosting site is, on average, 11 min after sunset, which varies with weather conditions. On a cloudy evening, bats left the roosting site earlier than usual (57). *Desmodus rotundus*, a species commonly studied for bat-borne viruses, becomes active before sunset (usually 20–30 min earlier), but *D. rotundus* in caves or old buildings start to fly out of the roost site after 15–25 min post-sunset (55). *Desmodus rotundus* are light-phobic, and their foraging time varies based on factors like season, lunar cycle, and weather conditions (58). In summer, *D. rotundus* leave their roost after 9 p.m., while in winter, they leave after 10 p.m. (58). Some reports suggest that 91% of *D. rotundus* are captured before 11 p.m., with only a limited number of bats captured after midnight (55, 59). *Desmodus rotundus* stays outside for 0.5 to 4 h a day, avoiding long flights after feeding (55). Male *D. rotundus* spend 1.5 h less outside than females. Capturing times usually range from 6 p.m. to 6 a.m. (60–64). To maximize captures, researchers should plan field sampling between the last and first quarter of the lunar cycle when *D. rotundus* is more active (58).

#### 3.1.2 Harp trap

Harp traps are made of lightweight aluminum line carriers and leg struts, combined with marine-grade stainless-steel frames (Supplementary material S7A) (65). Harp traps are placed across trails, small stream beds, or cave openings, with the first bank of lines allowing bats to either fall into a collecting bag immediately or pass through the first bank and become caught between the two banks before falling into the bag. Researchers sometimes modify harp trap materials based on availability and cost (65).

#### 3.1.3 Hand capturing

This technique is used to capture bats directly at their roosting site (e.g., cave or abandoned building). Bats are captured using long pole hand nets or directly by hand when bats have limited movement, such as in the mornings, to prevent injury, as they may become injured by the net (51, 62). Different hand capturing techniques are described below.

##### 3.1.3.1 Bucket trap

McCracken and Bradbury (66) used a plastic bucket cane to trap bats in caves. The opening of the cane was wide enough to encircle a cluster of specific bats, and then they bent the paddle to trap the bats. As bats cannot tangle in the plastic wall of the cane, they could not fly because the diameter of the bucket was smaller than the wingspan of the bats. Therefore, aluminum poles were used to extend and capture them, attached to the top of the cave. The bucket trap has some disadvantages, such as being heavy for the researchers, bats escaping the trap by walking along the wall, being potentially harmful to bats, and being difficult to remove bats from a bucket with a small diameter (66).

##### 3.1.3.2 Cone trap

Pérez-Torres et al. (67) modified the bucket trap using PVC pipes and mosquito nets, which gave it a cone shape (Supplementary material S8). They used a rope to close the opening, preventing bat escape, and another rope to secure the trap, making it easy to handle and capture large groups of bats in caves. We have used this method successfully for different species of bats.

### 3.2 Nets or trap monitoring and bat removal

To reduce stress on bats entangled in the nets, regular monitoring is required after setting the net or trap. Additionally, some bats may chew the net, make a hole, and escape. The team should check the nets at 15-min intervals (60, 68). When a bat becomes entangled in the net or trap, ideally, two individuals must be actively involved in removing the bats from the net immediately after a tangle. The first individual holds and untangles a bat using their left and right hands, while the second individual assists in removing the bats; experienced people can remove the bats alone (51). Bats should be removed promptly from mist nets to prevent stress, injury to delicate wing bones, and patagia. A badly tangled bat can be removed by cutting strands of the mist net to prioritize the welfare of the animal. In the case of a harp trap, one person removes bats from the harp trap, with another person accompanying them. When a team plans to set nets at resting sites of bats (e.g., cave or old building), several team members may be required to remove the bats, as many bats start to move or leave the resting sites at the same time and become entangled in the nets. All bats are securely wrapped in a sterile cotton cloth bag with drawstrings or ties at the top (Supplementary material S7C) (52, 69).

### 3.3 Field laboratory setup

The most crucial step after selecting a bat-capturing site is to determine the most suitable and safe capture site and laboratory setting. The laboratory setup should be conducted under the supervision of an experienced wildlife health researcher, particularly a veterinarian, to ensure standard procedures for animal handling and sample collection. To process the collected specimens and store them properly at the field site, maintain proper biosecurity and biosafety for both team members and captured bats. The goal is to enhance the laboratory environment for optimal performance and achievement of research goals with no risk of pathogen exposure to team members. To achieve these goals, several points should be followed.

#### 3.3.1 Location of the field laboratory

The place for the field laboratory where animals will be processed can be selected considering key logistic factors. Distance from the bat-capturing site, easy access, and a site away from the access or crowd of general people can facilitate the collection and transportation of samples. A site suitable for releasing bats should be close to the bat-capturing sites. Sites that are easy to clean and disinfect should be considered. From an animal welfare perspective, ideal sites should be safe for cloth bags containing bats to avoid carnivore attacks or drowning. The team should select a site where they can load and unload equipment easily. Radios are also needed to ensure that team members always have accessible communication.

#### 3.3.2 Organizing the bats

The team should identify a visible, accessible, and safe site to hang the cloth bags containing captured bats using a rope at a certain height to avoid dog, fox, or cat attacks. Bags should have enough distance to avoid contact between captured bats. The public should not have access to the bags or equipment. A tape with a biohazard icon can be used to demarcate the study area, and avoiding the presence of observers is encouraged to maintain biosecurity.

#### 3.3.3 Laboratory team setup

The team leader should outline laboratory activities, equipment, and logistics for specimen collection and processing at different stages of their work. The personnel assigned to each activity should understand their responsibilities. In our experience, for efficient processing, a minimum of four people is needed: (a) bat sampler, (b) bat restrainer or handler, (c) specimen or vial handler, and (d). data recorder or note taker (Supplementary material S9). The team is required to arrange and sort equipment on the laboratory table in accordance with the *a priori* defined protocol. If the number of bats captured is large, more people will be needed to work in a chain to quickly process and release bats at the site of capture. Each team member is required to sit in a designated place around the laboratory table to perform the sampling properly and smoothly (Table 2).

**Table 2:** Potential team organization for sampling in a field lab.

Person	Equipment needed
Bat sampler	Swab sticks, syringes, needles of different sizes, cotton balls, biopsy punch, bat marking tag set, measuring tape or ruler for morphometric measurement, weighing balance, pipette gun, pipette tips,
Bat restrainer	Not applicable
Specimen handler	Specimen collection tube or vials, scissors, cool or ice box, permanent marker, storage media (VTM, Trizol, etc.), Eppendorf tube, alcohol,
Data collector	Data collection sheet or questionnaire, pencil, eraser, permanent marker, species identification guidebook, camera

#### 3.3.4 Biosecurity and biosafety in the field lab

The team leader will ensure proper biosecurity and biosafety practices during laboratory work. For this, team members should have comprehensive training before the field expedition and a brief explanation on how to use appropriate PPE *in situ*. The team leader will ensure that team members maintain proper biosecurity and biosafety during the entire sampling period. Clean and disinfected equipment should be available at every step. Bags for biohazardous and waste materials should be easily accessible around the field lab bench. Separate polythene bags could be used for non-biohazardous waste materials to minimize biohazards.

#### 3.3.5 Disposal of biohazards

Team members must adhere to local environmental and health safety rules when storing or disposing of biohazards during fieldwork. Waste bags should remain in a secure location away from public movement. At the end of every day, the team should store the biohazard and waste bags in a secure and safe area. This area should be away from regular human access and any scavengers. The team should hand over all the biohazard bags to a designated authority for safe disposal as soon as possible or consider freezing or disinfecting the material when the work is done in remote areas with limited access to proper waste disposal.

### 3.4 Bat sampling

After the bat capture, cloth bags containing bats are brought to the field laboratory and hung in the designated space sequentially with enough distance to avoid contact between bats. To reduce bat stress and accelerate the release of animals during sampling, we propose a minimum of four members at the field laboratory bench (Supplementary material S9).

- *Bat sampler:* Collects and manages bat samples, including oral/rectal swabs, blood, biopsy, morphological measurements, microchip injection and confirmation, and monitoring the anesthesia of bats.
- *Bat restrainer or handler*: Removes the bat from the cotton bag followed by safe restraining and handling for sample collection. Induces and monitors anesthesia.
- *Specimen or vial handler*: Responsible for selecting, cutting, and placing swabs in a designated vial. Based on the research, swabs can be collected using Trizol, RNA later, Viral Transport Medium, or ethanol. Places vials sequentially in an ice box immediately after collection.
- *Data recorder or note taker*: This individual is responsible for maintaining a data sheet, recording all information related to bats, samples, and remarks, and identifying any missing samples or information collection.

### 3.5 Restraining

Proper restraining of bats is important to reduce the risk of accidents to team members (e.g., bites), as well as to minimize stress, pain, and the escape of bats, and to ensure the collection of quality samples. Bats can be restrained using chemical or physical methods, with handlers using leather gloves and double nitrile or latex gloves to prevent bites or scratches. There are different chemicals available to safely restrain bats. Table 3 offers an example of chemicals used to restrain *D. rotundus*. In the field, we have successfully used Isoflurane in a soaked cotton swab placed in a plastic container (e.g., Ziploc bag) with the bat for a few seconds with a dose of 0.1-0.2 mL (2 drops) (Figure 4). During physical restraint, the handler should be aware of stress and pain, and monitor respiration to prevent harming the animal (e.g., choking, fractures).

**Table 3:** List of chemicals reported to be used to restrain *Desmodus rotundus*.

Chemical name	Methods	Doses	References
Ketamine	Intramuscular	8.3–12.5 mg/kg	Streicker and Allgeier (63)
Chloroform	–	–	Tandler et al. (89); Matthias et al. (90)
Halothane	–	1.5 vol%	Schmidt et al. (91)
Sodium hexobarbital	Intraperitoneal	65 mg/kg	Schäfer et al. (92)
Hexobarbitone sodium	Intraperitoneal	0.065 mg/g	Kürten et al. (93)
Sodium pentobarbital	Intravenous, intubated, and ventilated with room air with a positive-pressure ventilator	35 mg/kg	Mellott et al. (94)

### 3.6 Biological sample collection

Team members should have a clear understanding of the type, amount, number of aliquots, collection method, and storing media of samples before starting the sampling. Here, we provide an overview of sampling methods.

#### 3.6.1 Oral or oropharyngeal swab

Restrainers use their left hand to restrain the bat’s body and wings, and their right hand to hold and maintain the bat’s head in an appropriate position so that sample collectors can easily collect oral or oropharyngeal swab samples (70). To open the mouth of bats, gently press behind the canine teeth of the bat with your thumb and index fingers. When dealing with small bats, it can be challenging to press with your thumb and index fingers while wearing thick gloves. The bat sampler can place a swab on the bat’s lips and observe its reaction. When the bat opens its mouth, hold it open with your thumb and index finger (Supplementary material S10). Based on the body and mouth opening size of the species, different swab sticks can be used. For example, for oral or oropharyngeal swab collection, standard foam swab sticks can be used, and for urine or urogenital swabs, a mini-tip swab would be more appropriate.

#### 3.6.2 Urogenital swab

For female bats, the urogenital swabs are directly collected from the vagina, and in the case of male bats, it is sometimes difficult to obtain a urogenital swab as their penis is narrow and the glans of the penis remains dry. Direct urine collection is recommended from male bats and any small-sized bat.

#### 3.6.3 Urine collection

There are two methods to collect direct urine samples from bats. (i) Spread a polythene sheet on the ground beneath the hanging bat bags. If bats urinate during the sample collection period, their urine will fall onto the sheet. Collect these urine samples separately for each bat directly from the sheet using a sterile syringe or micropipette, and transfer them to individual vials. (ii) Polythene bags are directly attached to the cotton bags containing the bats. If bats urinate during the sampling period, the urine is deposited in the polythene bag. Then, using a sterile syringe or micropipette, collect the urine and place it into the vials.

#### 3.6.4 Blood collection

**Figure 2:**
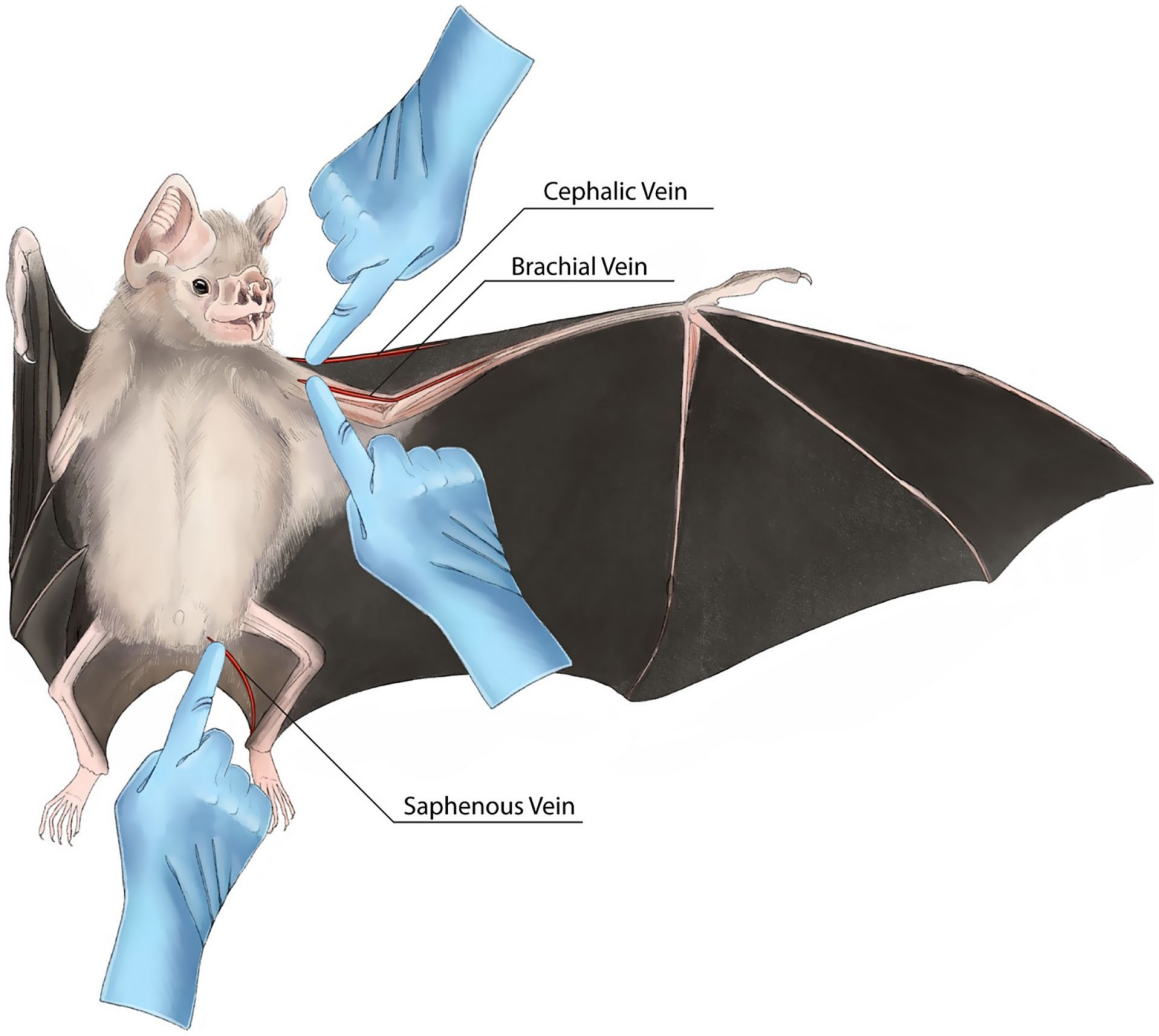
Different veins and blood collections sites to collect samples from a *Desmodus rotundus* bat. It is recommended to collect less than 10% of total blood volume or 1% of the body mass at a time from a bat (1, 52). In the case of small bats (e.g., *Desmodus rotundus*), either the brachial or cephalic vein along with recovering blood with a pipette is found to be more successful (1). For large bats (e.g., *Pteropus medius*), researchers can select either the cephalic or brachial vein to collect blood. In large bats, a saphenous vein is also selected for blood collection. Hand icons are used to indicate the area where pressure should be applied using a finger to facilitate needle insertion and blood flow.

Blood samples can be collected from a bat from different sites, including the brachial, cephalic, and saphenous veins (71) (Figure 2). Some researchers have collected blood directly from the heart of bats, which should be done only in anesthetized animals when conducting lethal sampling (72). Needles of different sizes can be used (e.g., 25 to 29 gauge) depending on the species of bat, sites of bleeding, and the experience of the researcher. Collecting blood directly from the vein using a syringe and needle is difficult and can cause hematoma. To minimize stress on bats, veins are pinched, and blood is collected using a pipette (1) (Supplementary material S11). A 75 μL glass hematocrit tube can also be used to collect blood. The amount of blood should not exceed 1% of the bat’s weight or 10% of the total volume of blood (1, 52).

#### 3.6.5 Rectal swab or feces collection

Rectal swabs are collected directly from the rectum using sterile swab sticks (70, 73). To reduce stress on the bat, a lubricating ointment can be used, such as soaking the swab stick in viral transport medium (VTM) before inserting it into the rectum. To collect direct feces, check the cloth bag; if fresh feces are available in the bag, collect them using a sterile swab stick or forceps and place them in the vials.

#### 3.6.6 Biopsy samples

**Figure 3:**
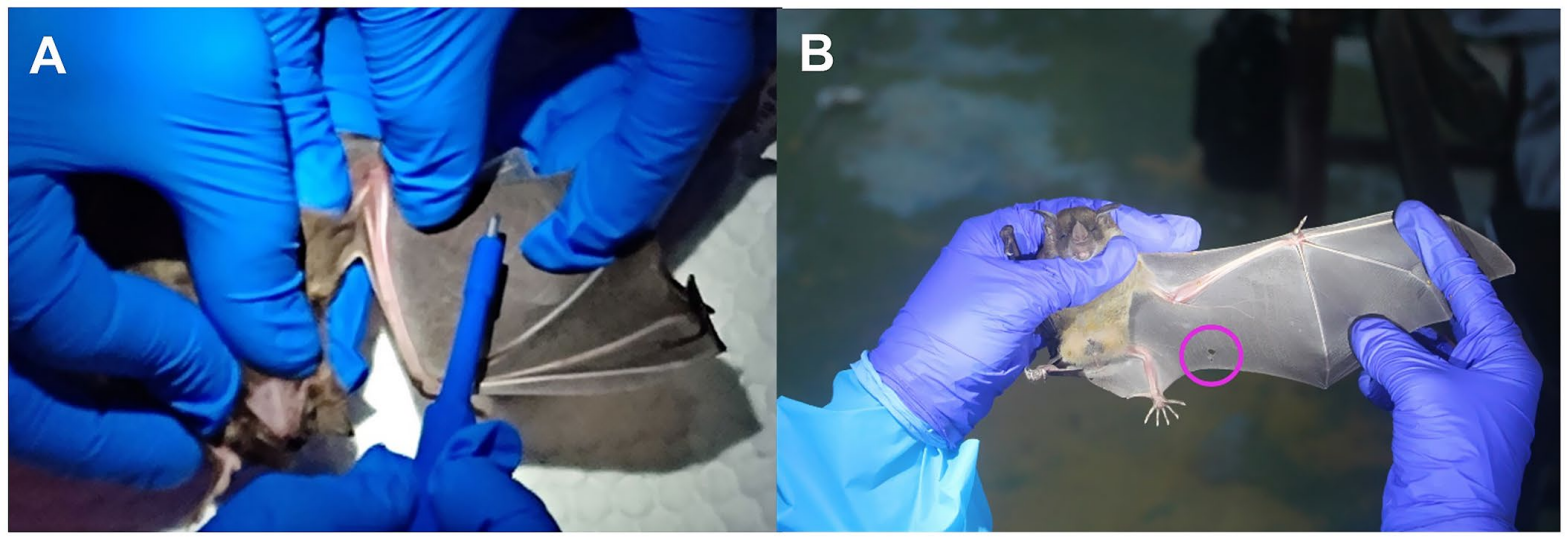
Collection of wing biopsy specimens from a bat. **(A)** Site selection, preparation, and biopsy specimen collection from a bat using a biopsy punch. **(B)** Observing the hole created on the wing due to the collection of a2×2 biopsy from a bat’s wing. Photo: Paige MacClure and Carlos Bravo.

Biopsy samples are collected for bat species identification and other purposes, such as population genetics. Hair or wing samples are collected as less invasive methods. To collect the wing biopsy, select an area in the plagiopatagium that has no major blood vessels and collect the skin using a biopsy punch nearest to the edge of the wing membrane. Try to avoid punching the center of the wing membrane (Figure 3A). Spread the wings of a bat against a plain, hard object. Disinfect the area that will be punched with an ethanol pad. Gently press the punch on the selected area of the wings to collect the biopsy and place it in a 70% ethanol solution or silica gel (Figure 3B). In the case of hair sample collection, a long and densely hairy area is selected; primarily, hair samples from the back of the neck are collected using scissors.

#### 3.6.7 Organ or tissue sample collection

Researchers collect different organ samples from euthanized bats, typically 200 milligrams (mg) of liver, lung, kidney, spleen, intestine, and brain. The type and number of organs collected are based on research purpose, resource availability, and sample collection permission (74). To collect organ samples, individual bats are placed on a clean absorbent sheet (e.g., polythene sheets are helpful in this case), which is replaced for each individual bat to reduce cross-contamination (75). The heart and lungs should be collected first to minimize contamination (e.g., intestinal content) by opening the sides of the thorax using scissors. To collect the liver, kidney, spleen, and other organs located in the abdominal cavity, the abdominal cavity is opened with scissors. Desired organs are removed from the cavity, and the required number of organs is collected. Researchers must use a separate set of scissors and forceps to collect organ samples from different bats. Additionally, to collect different types of organ samples from a single bat, different forceps and scissors should be used to avoid cross-contamination from one organ to another. This is particularly important if organ tropism studies are to be conducted. If resources are limited, researchers should at least decontaminate their instruments by removing excess tissue using disposable paper or gauze, then introduce the instruments into clean ethanol and flame them in an alcohol lamp between the collection of two different bats or organs from a single bat. This practice is critically important when researchers try to determine the spread of a pathogen within a single host and investigate the breadth of affected organs due to different organisms. If the purpose of the research is to investigate the pathological changes in different organs due to the infection of a certain organism, further caution should be followed during organ sample collection. During the selection of an area of an organ to collect specimens, researchers should aim to cover both healthy and infected areas of the organ that may be grossly visible. This will help to identify the differences and changes due to infection (75).

#### 3.6.8 Serum

Obtaining sufficient serum is always challenging due to the vein distribution and size of bats. To obtain high-quality serum samples, transfer blood into a vacutainer with a serum separation gel tube immediately after blood collection. Gently turn the tube upside down and vice versa, and place the tube into a cooler (4–8°C) where it fits the rack/holder to keep it in an upright position for about 5–6 h. To start serum separation using a centrifuge, remove the vacutainer tube from the cool box and keep it at room temperature for 15–20 min. Place the tube into the machine and set it to 5,000 RPM (rotations per minute) for 5 min. The centrifuge will stop automatically; wait until it has completely stopped rotating. Examine the vacutainer tubes and assess the serum quality, checking whether it is clear and transparent. If the serum is not transparent enough, re-centrifuge it. Using a micro-pipette with appropriate tips, collect the serum from the upper part and place it into clean transparent vials, which should be external screw-cap tubes with gaskets. The vacutainer tube with all the remaining contents should be placed into a biohazard bag if it is plastic, or a sharps container if it is made of glass. The serum samples are then stored in a dry-ice cooler, or a liquid nitrogen tank if the processing was conducted in the field, or an ultra-low freezer until further laboratory tests.

### 3.7 Euthanasia

**Figure 4:**
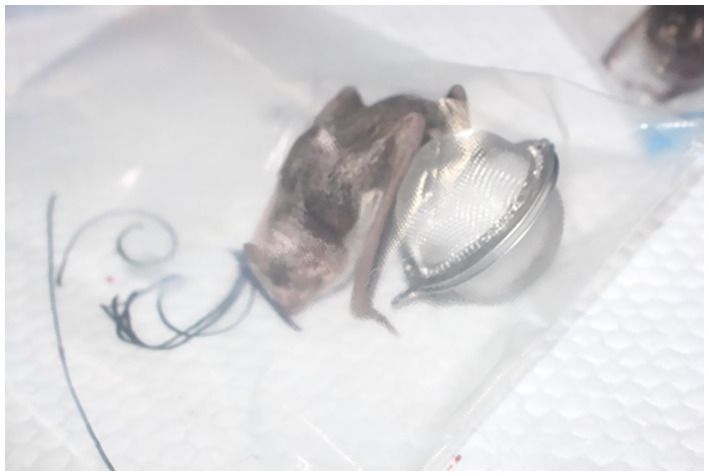
Anesthesia of a bat in a Ziploc chamber bag using isoflurane. Inside the Ziploc bag, a stainless-steel mash tea ball contains a cotton ball soaked with isoflurane. Photo: Luis E. Escobar.

Researchers may need to perform euthanasia on bats that are injured (51). Researchers may also collect a representative sample of animals captured to deposit their carcasses and skins for further examination and study (voucher specimens). Lethal sampling, however, must adhere to local laws and regulations regarding euthanasia and lethal sample collection. Usually, a veterinarian directly performs euthanasia, following animal welfare and biosecurity guidelines, specifically the “American Veterinary Medical Association (AVMA) Guidelines for the Euthanasia of Animals” (latest edition) and the “Guidelines for Euthanasia of Nondomestic Animals” of the American Association of Zoo Veterinarians (76). In special situations, personnel trained by veterinarians can perform the euthanasia of bats in the field. The protocol for field euthanasia involves administering an overdose of isoflurane gas anesthesia, followed by cervical dislocation of the bat (77), as described by the United States Geological Survey-approved euthanasia methods for small bats (Microchiroptera) (78). First, use a Ziploc bag as a chamber and a stainless-steel mesh tea ball infuser with a ball of cotton containing 0.5–1.0 mL of isoflurane for a 500 mL volume chamber (79) (Figure 4). Bats are then introduced into the sealable bag and remain there until no respiration is observed or detected by the veterinarian. If isoflurane fails to euthanize the bat, cervical dislocation is performed by applying pressure to the neck to dislocate the spinal column from the skull (76). Nevertheless, performing cervical dislocation on bats could expose researchers to the rabies virus (80). As an alternative to cervical dislocation, researchers can consider the use of carbon dioxide and exsanguination of an anesthetized or already unconscious bat.

### 3.8 Samples labeling and aliquots

Identification and labeling are crucial steps for successful sample collection, laboratory testing, data analysis, and interpretation. Inaccurate labeling and misidentification of specimens can result in project failure, incorrect lab results, inappropriate data analysis, and misleading interpretation. Therefore, we recommend identification numbers at two different stages. First, assign a specific identification number for a bat as an individual. In the second stage, assign a specific identification number for each specimen collected from an individual bat. For example, if a team decides to capture bats in Argentina, they can start the identification number as A0001 for their first bat. The team will collect and record all the information or data (e.g., age, sex, body condition, and different measurements of that bat) under the identification number A0001 (Table 4). In the second stage, the team can use a syntax that describes the specimen type and storage media used. For example, if a team collects an oropharyngeal swab and stores the swab in Trizol, they can assign the identification number of the specimen as A0001.OPT. Here, A0001 refers to the individual bat number from which this specimen originated, OP means oropharyngeal swab, and T means Trizol. Similarly, A0001.BSN indicates blood serum with no media from the bat identified as A0001. Sometimes, the label of specimens is lost or erased for various reasons. In that situation, it is difficult to trace back the specimen’s source and identification. Without labeling, a specimen becomes useless and should be discarded in the interest of the rigor of the study.

**Table 4:** An example of individual bat and specimen identification number generation.

Bats identification number			Sample identification number		
Location identification (first two or three digits)	Animal type	Numeric number for the sequence	Specimen type	Storage media	Specimen identification number
COL for Colombia	B for bat.	0001 for the first bat	OP for oropharyngeal swab	T for Trizol	COLB0001. OPT
				L for Lysis buffer	COLB0001. OPL
				V for VTM	COLB0001. OPV
				N for No media	COLB0001. OPN

### 3.9 Bats species identification and tagging of individuals

To identify bat species, it is necessary to have bat species identification guides during field sampling (Supplementary material S12). For this, different morphological measurements such as weight, head, nose-leaf, body, tail, forearm, and hind foot length are collected from captured bats and compared with the previously reported species to determine species (Supplementary materials S12B, S12C) (51). Furthermore, researchers use different temporary and permanent marking systems to monitor bats and avoid resampling during a single sampling event, as well as to follow up on a specific bat at different time intervals. The recommendation is to avoid permanent marking of bats for successful research if a temporary marking system can be employed. For temporary marking, people use clipping of dorsal fur from different body areas and pinpricking the wing membrane to create unique tattoos for bats (57). For permanent marking of bats, light tags, wing bands, and microchips can be used (51). Different kinds of microchip injectors and scanners are available in the market for use in bats. Microchips are injected subcutaneously by pulling up the skin on the back of the bat to insert the needle with the Passive Integrated Transponder (PIT) tag under the skin. Then, the PIT tag is ejected, and the needle is pulled out gently (81, 82). Finally, a drop of surgical glue on the injection point in the skin is recommended to secure the tag after the release of bats back into their habitat. Each tag will have a unique number to identify each individual bat and avoid resampling.

## 4 Post-field processing and quality control

### 4.1 Specimen processing, transportation, and storage

#### 4.1.1 Complications with labeling

##### 4.1.1.1 Loss of label

There are some commercially available labels that are not suitable when you plan to store the samples in liquid nitrogen. Some adhesive glues do not work as they should in liquid nitrogen. Besides, frequent handling of specimens can damage these labels.

##### 4.1.1.2 Erase of label

Using a permanent marker to label vials can result in the loss of the label when vials are in contact with alcohol or Trizol. There are commercially available water- and solvent-proof labels with good adhesive capacity that do not lose quality in liquid nitrogen. Alternatively, adding a layer of transparent scotch tape may help protect the label.

#### 4.1.2 Temperature maintenance

Temperature maintenance is crucial for ensuring high-quality samples and accurate results from laboratory testing (83). The time between sample collection and storage, and between samples brought out from storage to testing, is critical. Samples and their containers or boxes should be marked and organized carefully for easy location to avoid unnecessary thawing of the specimens and to achieve optimal results. Storage temperature and duration depend on factors like research objectives, the distance between field sites or primary storage facilities and the final laboratory where specimens will be tested, targeted pathogens, available facilities, lag time between collection and testing, and storage and transport media. Different methods and storage facilities can be used to store the specimens in different situations (19, 83).

##### 4.1.2.1 Short-term storage

The term “short” refers to the ability of the cooling materials to maintain a specific temperature without losing the viability and integrity of the sample (84). Dry ice or a cool box are examples of short-term storage.

###### 4.1.2.1.1 Cool box

Specimens are stored in cool boxes with ice blocks, frozen ice packs, or frozen gel packs. Samples should be transferred to liquid nitrogen, an ultra-low freezer (−70°C or below), or dry ice containers within 6 h of being stored in the cool box (19, 83). Ice and gel packs should be frozen for at least 12 h at −20°C or −70°C. The temperature of the cool boxes should be monitored periodically, with ice packs being replaced every 10–12 h. If the environmental temperature is high, ice packs need to be replaced more frequently (e.g., every 3–6 h).

###### 4.1.2.1.2 Dry ice (−78.5°C)

Dry ice is used for specimen storage in a properly sealed insulated container, allowing for 12 h of ultra-low temperature maintenance. The duration of storage depends on the external temperature. Dry ice sublimes quickly at room temperature and needs to be replenished or added every 12 h (85). Dry ice is commonly used for sample transfer/transport from remote areas where liquid nitrogen or ultra-low freezer facilities are lacking.

##### 4.1.2.2 Long-term storage

Specimens are stored long-term for archiving and future laboratory testing. An ultra-low temperature freezer or liquid nitrogen are the choices. In the case of liquid nitrogen, the amount of liquid nitrogen available in the container is checked regularly and needs to be refilled when the level is near the top of the racks or tube holders.

###### 4.1.2.2.1 Ultra-low freezer (−80°C)

Biological samples can be stored for many years in an ultra-low freezer (19). Ultra-low freezers require continuous temperature monitoring and evaluation. A temperature log sheet is attached to the door of each freezer, and a designated person records the temperature of the freezer in the log sheet at a certain time interval (generally every 12 h). More sophisticated facilities have implemented real-time temperature monitoring systems that use sensors placed inside freezers to send temperature readings to central computers and communication centers, so laboratory managers are notified immediately if significant temperature fluctuations occur (86). Researchers must ensure a continuous supply of electricity or backup generators for the ultra-low freezer containing samples, as a power shortage may cause the freezer temperature to drop and potentially degrade samples.

###### 4.1.2.2.2 Liquid nitrogen container (−196°C)

Samples can be stored in a liquid nitrogen container with secure closures for extended periods (19). Researchers need to continuously monitor and refill the container, which should be refilled at about 10-day intervals or earlier based on the container’s condition and quality, usually when the level is near the top of the racks or tube holders.

### 4.2 Cleaning and disinfecting

All the field equipment and clothes used during bat capturing and sampling become contaminated with the biological materials of bats. Contaminated equipment and the laboratory environment pose a major source of infection for both team members and bats. Researchers need to spray disinfectants on the tables to clean them and use alcohol on their hands to prevent cross-contamination after each bat sampling. After completion of everyday fieldwork, non-disposable equipment and laboratory floors should be cleaned and disinfected using chlorine bleach or Amphyl (hospital-grade Lysol) (52). A list of reagents that can be used for different equipment is listed in Supplementary material S3.

### 4.3 Waste management and disposal

Biohazards are substances that pose a significant threat to human, animal, or environmental health due to their potential for infectious or dangerous effects (87). Improper disposal of biohazardous material may create public outrage, spread infections, and attract other animals (88). Hence, potentially infectious biological wastes, including body fluids, carcasses, and bedding materials, are considered hazardous or nonhazardous when they come into contact with animals or humans (87, 88). A comprehensive biological waste management and disposal plan is essential for effective biosafety and biosecurity practices in bat sampling. Before going to the field, consider all the options you must follow for waste management guidelines.

#### 4.3.1 Non-sharp biological waste

Biological waste, except needles and glass, is placed or deposited in biohazard bags. A biohazard bag should not be filled to exceed two-thirds of its capacity. A filled biohazard bag is sealed properly without pressing it by hand or foot. After sealing the bags, quaternary ammonium compounds (for example, alkyl dimethyl benzyl ammonium chlorides) are spread over the biohazard bag. Place the sealed and sprayed biohazard bags in a secure location away from human and animal movement.

#### 4.3.2 Sharp biological waste

Needles and glass are placed in a sharps container with quaternary ammonium compounds (¾ filled) to decontaminate the sharp materials. After completion of fieldwork at a certain site, the safest disposal of biological waste is to hand over waste to the designated biological waste disposal authority or institution in that area for disposal. The team leader may arrange collaborations with a nearby health facility for safe and immediate disposal of waste materials.

### 4.4 Specimens’ shipment

Sometimes, researchers need to ship samples from one country to another. During this shipment process, there are different rules and regulations that researchers need to follow. For example, shipping specimens collected from a certain species of bats to the United States requires compliance with guidelines and permits established by the Convention on International Trade in Endangered Species of Wild Fauna and Flora and the Animal and Plant Health Inspection Service of the U. S. Department of Agriculture. Import or export permits vary based on the bat species, types of specimens, source of origin, and the targeted destination of the shipment. Obtaining all necessary permits is often simple, but understanding the process may take time and effort. Therefore, it is wise to be prepared and start the application process for a permit at least six to four months before shipping. All permits need to be included with the specimens’ package during transportation.

In the meantime, researchers should explore and identify a suitable shipping agency to ship the specimens. A shipping company also needs additional documents for the shipment based on its company policy. Before or during the signing of the contract for the specimen’s shipment, carefully and clearly explain the transportation requirements (for example, the need to always maintain the cold chain) to uphold the standards of quality specimens during shipment. Shipping agencies, however, generally fail to sustain the cold chain and adhere to schedules (e.g., World Courier, *Escobar* per. Comm. 1/8/2025). A monitoring report at the end of the shipment process could help confirm if the temperature chain was maintained.

## 5 Conclusion

A systematic plan is crucial for the successful, ethical, and safe collection of high-quality biological samples (19). In this situation, different factors should be considered, including targeted species, sampling methods, equipment and supplies, local legislation, and personnel health hazards. We recognize that bat research spans a vast spectrum of ecological and logistical contexts, ranging from well-resourced laboratories in urban settings to resource-limited areas with challenging terrain and minimal infrastructure. For instance, collecting and storing samples can be particularly challenging in resource-limited countries, where access to specialized equipment and trained personnel may be difficult or unavailable. Therefore, prioritizing the availability of alternative options and careful planning is crucial in overcoming any challenges. For instance, in the event of a limited supply of dry ice, ice may be used as an alternative to maintain the cold chain. Specimens should then be transferred to a freezer or placed on dry ice as soon as it becomes available. Similarly, during field laboratory procedures, if the recommended number of four personnel is not attainable due to constraints such as a lack of trained staff or limited resources, responsibilities should be redistributed among the available personnel to ensure the successful completion of research objectives while maintaining proper biosecurity and biosafety.

Our framework is built upon fundamental principles of biosecurity, biosafety, and animal welfare, which remain essential regardless of the operational environment (Figure 1). Prioritizing biosecurity and biosafety is essential for both raising awareness and enhancing capacity. The core principle of minimizing exposure to pathogens requires researchers to creatively implement appropriate barriers within their available resources. In resource-limited settings, where the risk of disease emergence is often highest, the adaptability of field protocols is critical. National and international organizations should promote guidelines for biosafety, biosecurity, and animal welfare practices in bat research. This guide is not exhaustive but hopefully provides a framework that supports rigorous, safe, and ethical research worldwide. By understanding and strategically adapting this guide to the unique ecological and logistical realities of diverse field settings, researchers can enhance the quality and comparability of data. Standardized methods and data could help advance our collective efforts in understanding and mitigating bat-borne pathogen risks while safeguarding bat populations.
