# Supplementary material for: A field guide for sampling bats (Chiroptera) for eco-epidemiological studies
Source: Front Vet Sci. 2025 Sep 9;12:1605150. doi: 10.3389/fvets.2025.1605150 (PMC12456185; doi:10.3389/fvets.2025.1605150)
Supplement: Supplementary file 1 [file Supplementary_file_1.docx]

**Supplementary 1:** An example of categorization of risks based on the surrounding environment (sampling locations), bat species, and filed operation procedures

***1. Operational Risks***

| **Risk Factor** | **Low Risk** | **Medium Risk** | **High Risk** | **Mitigation Measures** |
| --- | --- | --- | --- | --- |
| Team Experience | Highly experienced | Mixed experience | Inexperienced | Training, supervision |
| Equipment Access | Full equipment | Limited backup | Minimal equipment | Equipment checklist |
| Medical Access | <30 min | 30-60 min | >60 min | Emergency protocols |

***2. Environmental Risks***

| **Risk Factor** | **Low Risk** | **Medium Risk** | **High Risk** | **Mitigation Measures** |
| --- | --- | --- | --- | --- |
| Cave Environment | Well-ventilated, multiple exits | Limited ventilation, few exits | Poor ventilation, single exit | Ventilation equipment, buddy system |
| Weather Conditions | Clear, mild | Rain, moderate temps | Extreme temps, storms | Weather monitoring, postponement protocols |
| Terrain | Easy access, flat | Moderate slopes | Steep, unstable | Safety equipment, route planning |

***3. Biological Risks***

| **Risk Factor** | **Low Risk** | **Medium Risk** | **High Risk** | **Mitigation Measures** |
| --- | --- | --- | --- | --- |
| Bat Species | Non-aggressive, small | Moderate size | Large, aggressive | Species-specific protocols |
| Known Pathogens | Few documented | Several known | Multiple high-risk | Enhanced PPE, vaccination |
| Colony Size | Small (<100) | Medium (100-1000) | Large (>1000) | Adequate staffing, ventilation |

**Decision Matrix**

- Low Risk: Standard protocols
- Medium Risk: Enhanced protocols, additional supervision
- High Risk: Full containment protocols or abort mission

**Required Actions**

1. Complete assessment before each field session
2. Document all risks and mitigation measures
3. Review with entire team
4. Establish go/no-go criteria
5. Regular reassessment during operations


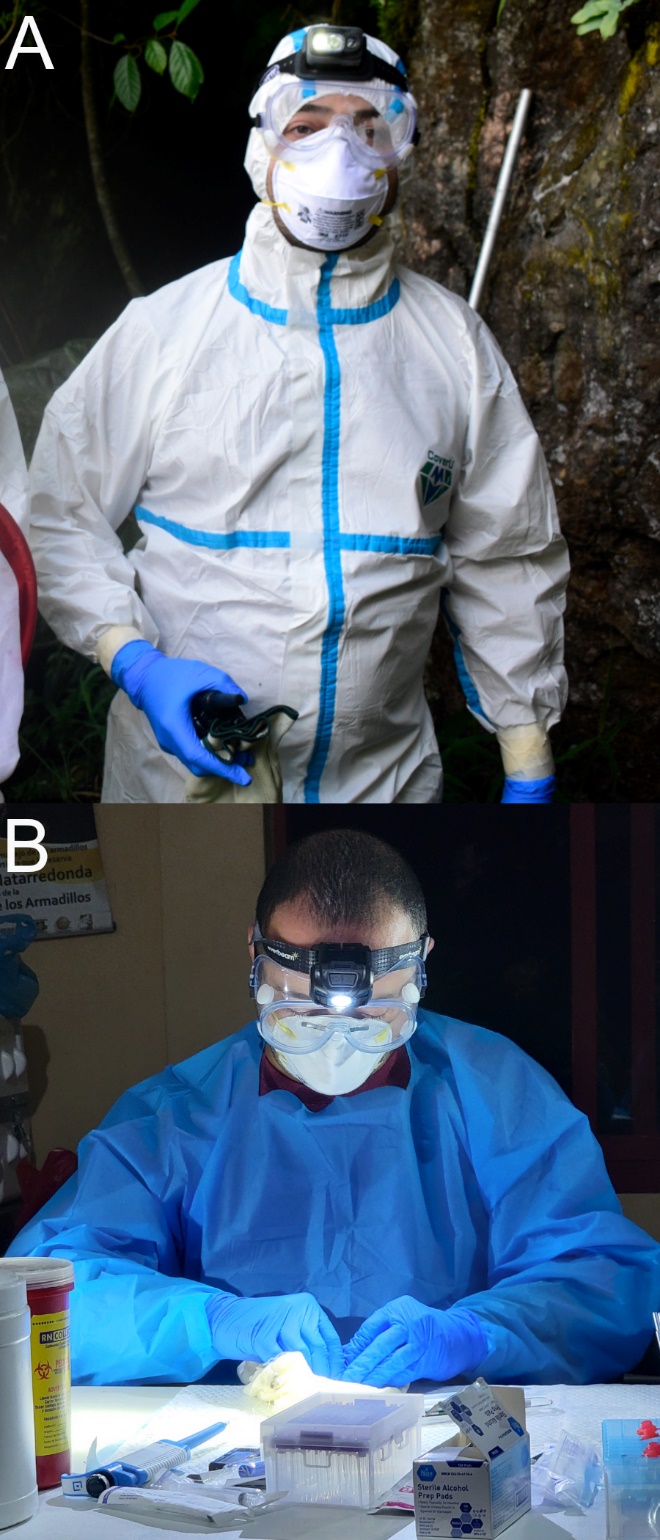


**Supplementary 2:** Use of different personal protective equipment (PPE) based on the settings of bat capturing and handling. A. Wearing coverall, N95 respirator, protective googles, nitrile gloves and securing the gloves and coverall at wrist using masking tape to avoid exposure of body parts before entering the cave. B. Wearing blue gown along with other components of the PPE during specimen collection in the field laboratory.

**Supplementary 3: Field Decontamination Procedures**

- 1. **Personnel Decontamination**

1. **Level 1 (Routine)**
2. PPE Removal Sequence
   - Outer gloves
   - Face shield
   - Coveralls
   - Inner gloves
   - Respirator
   - Hand hygiene
3. **Level 2 (High Risk)**
4. Primary Decontamination
   - Gross contamination removal
   - Chemical disinfectant application
   - Rinse procedure
5. Secondary Decontamination
   - PPE removal in designated area
   - Full body shower if available
   - Fresh PPE protocol
   1. **Equipment Decontamination**
      1. **Field Equipment**

| **Equipment Type** | **Disinfectant** | **Contact Time** | **Method** | **Frequency** |
| --- | --- | --- | --- | --- |
| Mist nets | 10% bleach | 10 minutes | Immersion | After each use |
| Holding bags | Virkon 1:200 | 10 minutes | Immersion | After each use |
| Instruments | 70% ethanol | 5 minutes | Wipe/Spray | Between bats |
| Electronics | Approved wipes | 2 minutes | Wipe | Daily |

- - 1. **Laboratory Equipment**

1. Processing Area
   - Initial cleaning
   - Disinfectant application
   - Contact time monitoring
   - Final rinse
   - Documentation
2. Storage Equipment
   - Temperature monitoring
   - Regular cleaning schedule
   - Maintenance protocol
   - Contamination monitoring

**Supplementary 4:** A preliminary list of items of first aid required during field work

- First aid kit
- Antivenin (if applicable)
- Emergency contact list
- Satellite phone
- GPS device

**Supplementary 5**: An exemplary emergency response protocol

- 1. **Exposure Incidents:**

1. ***Bat Bite or Scratch Protocol***
2. Immediate Response
   - Stop all activities
   - Wash wounds thoroughly (15 minutes with soap and water)
   - Apply antiseptic solution
   - Document incident (time, species, circumstances)
3. Medical Response
   - Contact supervisor immediately
   - Activate emergency medical protocol
   - Transport to designated medical facility
   - Initiate post-exposure prophylaxis assessment
4. Follow-up Actions
   - Complete incident report
   - Review of protocols for potential improvements
   - Update team training if needed
5. ***Aerosol Exposure Protocol***
6. Immediate Actions
   - Exit exposure area immediately
   - Remove and contain contaminated PPE
   - Shower if facilities are available
   - Document exposure details
7. Medical Assessment
   - Contact occupational health
   - Monitor for symptoms (14-day minimum)
   - Follow institutional exposure guidelines
   1. **Environmental Emergencies**
8. ***Cave/Confined Space Emergency***
9. Emergency Exit Protocol
   - Maintain buddy system
   - Follow marked exit routes
   - Use emergency air supply if available
   - Activate emergency beacons
10. Team Response
    - Headcount at assembly point
    - Contact emergency services
    - Deploy rescue equipment if trained
    - Maintain communication protocol
11. ***Severe Weather Protocol***
12. Risk Assessment
    - Monitor weather conditions
    - Establish trigger points for evacuation
    - Secure equipment and samples
    - Protect captured bats
13. Evacuation Procedures
    - Predetermined rally points
    - Equipment priorities
    - Sample preservation protocol
    - Team accountability system
    1. **Medical Emergencies**
14. Life-threatening Emergencies
    - Immediate evacuation protocol
    - First aid implementation
    - Emergency services contact
    - GPS coordinates ready
15. Non-life-threatening Emergencies
    - Assessment protocol
    - First aid implementation
    - Transport decision tree
    - Documentation requirements

**Supplementary 6:** An example of equipment is required during capturing bats

- Mist Nets / harp traps/ cone traps
- Nets (various sizes)
- Poles
- Guy lines
- Stakes
- Repair kit
- Clothe bags (multiple sizes)
- Leather gloves
- Measuring tape
- Machete
- Headlights


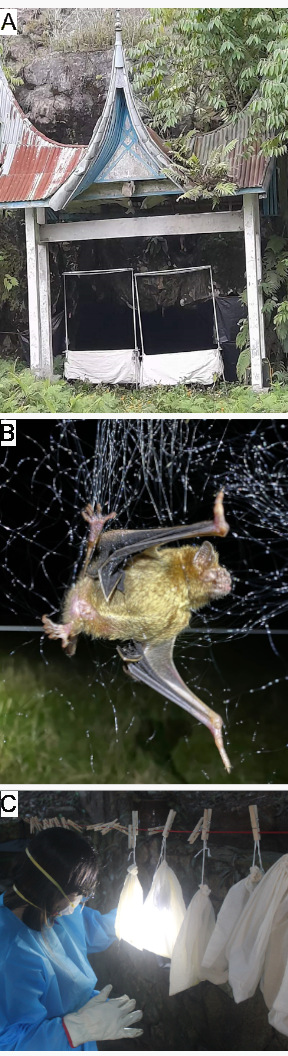


**Supplementary 7:** Mist-net or harp trap setting and monitoring, removal of bats and safely storing the captured bats in cloth bags. A. Setting harp trap in an abandoned house (Photo: Alice C. Hughes). B. A bat entangles on the mist net (Photo: Luis E Escobar). C. Placing the captured bats into the cloth bags and hanging the bags in a secure place (Photo: Luis E Escobar).


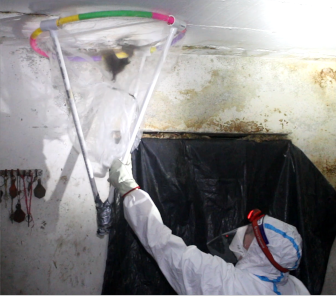


**Supplementary 8: Bat capturing methods.** Researchers use the cone trap to capture bats from the roof of an abandoned building. Photo: Paige Van de Vuurst.


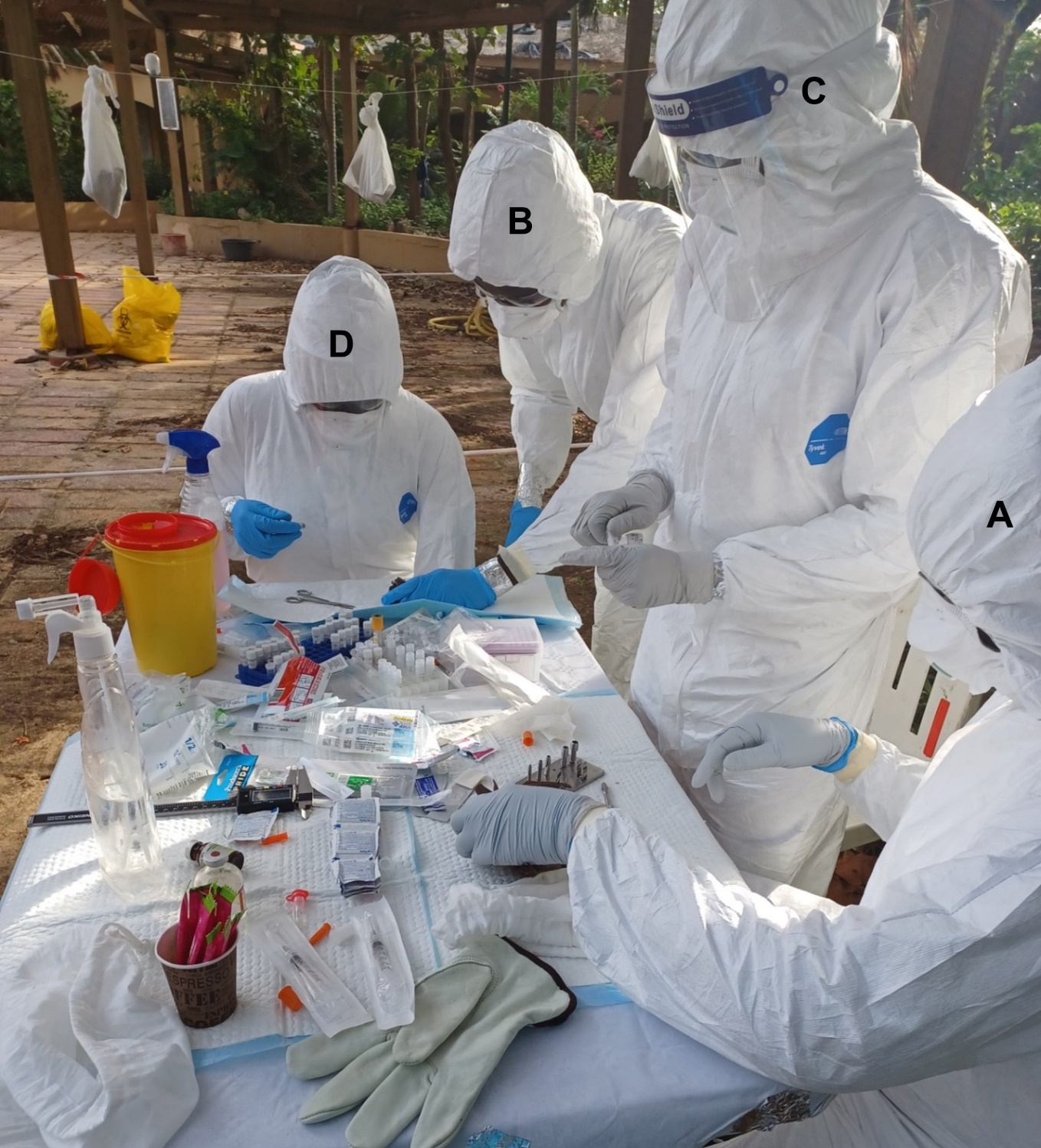


**Supplementary 9: Laboratory team setup at field for bat sampling.** Potential team structure during bat sampling. A. Bat sampler. B. Bat restrainer or handler. C. Specimen or vial handler. D. Data recorder or note taker. Here, the bat handler is checking and confirming the information recorded by the data collector after completion of sample collection from a bat. Photo: Luis E Escobar.


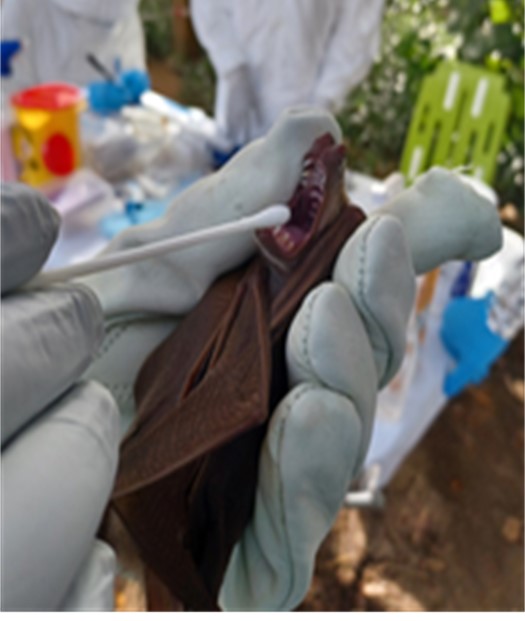


**Supplementary 10:** Restraining of a bat and collection of oral swab samples from bats. Bats were restrained with the left hand of the researcher and gently pressed behind the canine teeth of bat with thumb and index fingers to open the mouth followed by collection of swab samples using swab stick.


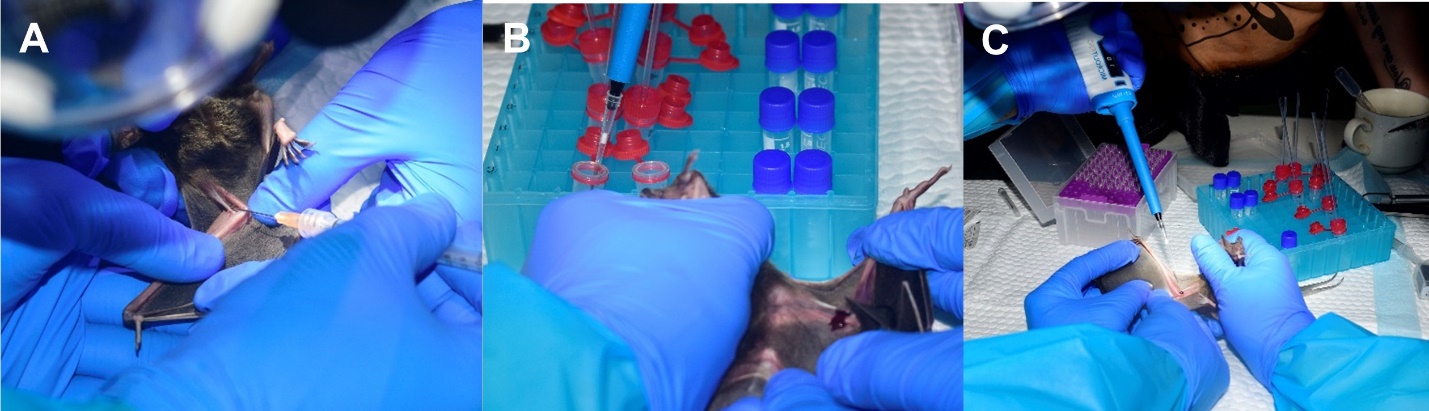


**Supplementary 11:** Collection of blood from the brachial vein of a bat. A. Venipuncturing the brachial vein using a needle. B. Drops of blood accumulated outside the vein. C. Collection of drained out blood using a pipette. Photo: Luis E Escobar.


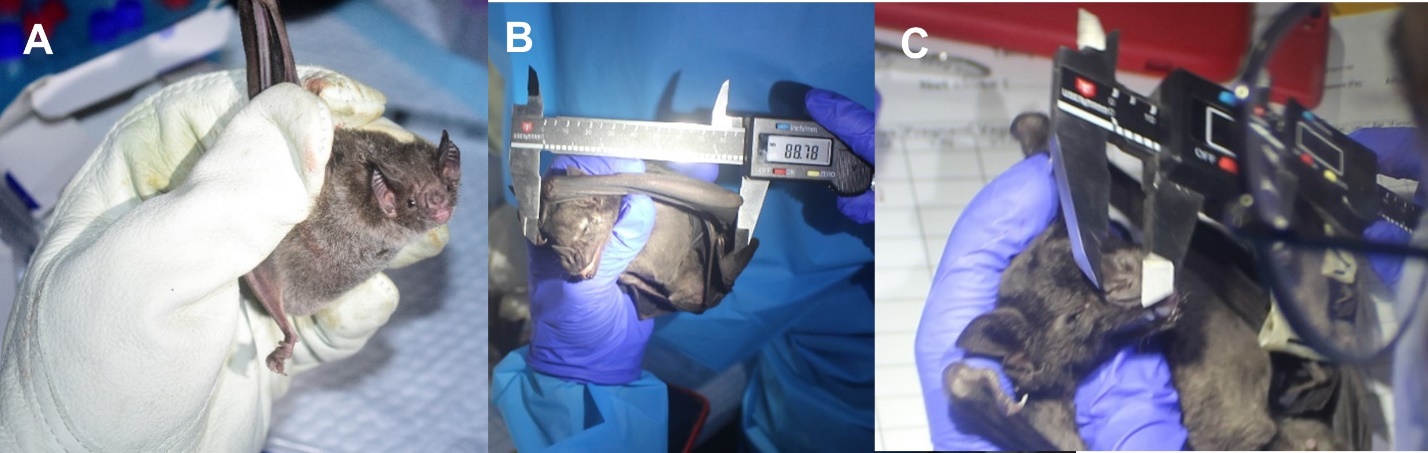


**Supplementary 12:** Identification of a bat based on its morphological features following standard bat identification guidelines. A. Observation of a Desmodus rotundus bat from different sites to get a clear idea about the features of the bat. B. Measuring the forearm of a *Carollia perspicillata* bat using slide calipers. C. Measuring the nose-leaf length of a *Carollia perspicillata* bat using slide calipers. Photo: Luis E Escobar.


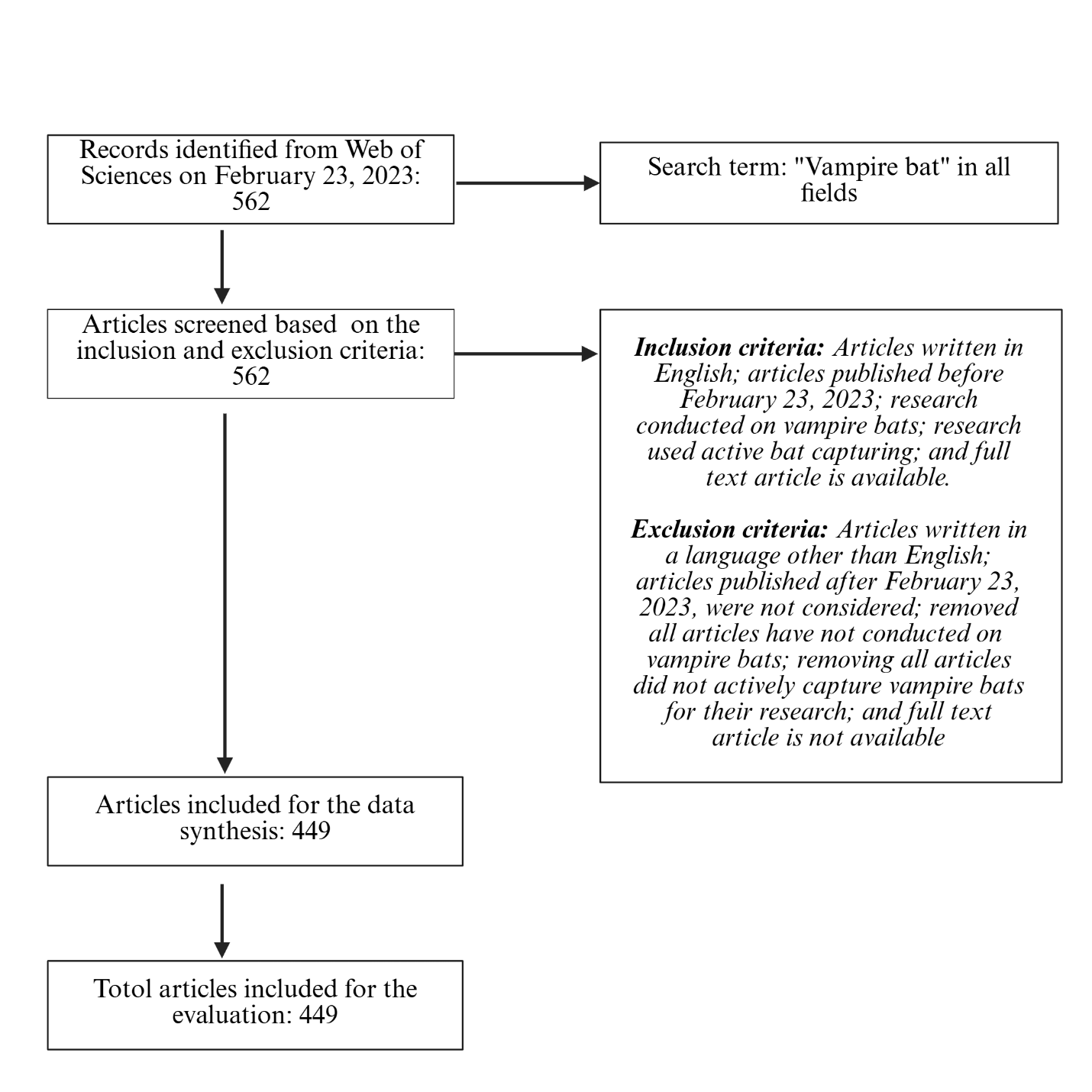


**Supplementary 13**: Identification of studies published in the Web of Science
